# Exploring the psychological dynamics of intensive smartphone use among university students: a phenomenological study

**DOI:** 10.3389/fpsyg.2026.1953912

**Published:** 2026-10-07

**Authors:** Tuğba Türkkan, İbrahim Yücel

**Affiliations:** Department of Social Work, Faculty of Health Sciences, Gümüşhane University, Gümüşhane, Türkiye

**Keywords:** digital behavior, intensive smartphone use, phenomenology, psychological dynamics, university students

## Abstract

**Background:**

Intensive smartphone use is common among university students, yet existing research has predominantly focused on usage duration, behavioral indicators, and quantitative correlates. Less is known about how students subjectively experience intensive smartphone use and the emotional, cognitive, social, and contextual meanings they associate with aspects of use that they perceive as difficult to regulate or problematic.

**Methods:**

This qualitative study employed a phenomenological design. Twenty-eight university students reporting at least 4 h of daily smartphone use were recruited using purposive and convenience sampling. Data were collected through semi-structured interviews complemented by 14-day micro-journals and analyzed using thematic analysis.

**Results:**

Five themes were identified: (1) relationship with the smartphone and perceived control, (2) coping functions of smartphone use, (3) content and social feedback, (4) self-evaluation, and (5) daily use patterns and effects. Participants described smartphone use as closely intertwined with emotional regulation, cognitive escape, social interaction, social-media-based feedback and comparison, self-evaluation, and routinized patterns of everyday use. Their accounts also indicated that some experiences perceived as difficult to regulate occurred within broader patterns of intensive smartphone use, while social-media-specific processes were particularly salient for many participants.

**Conclusion:**

The findings highlight interrelated emotional, cognitive, social, and behavioral dimensions of intensive smartphone use and suggest a potentially cyclical pattern that should be understood as a theoretical interpretation of participants’ lived experiences rather than as an established causal mechanism. The study underscores the importance of examining the meanings and functions of smartphone use beyond screen time alone.

## Introduction

With the rapid advancement of the internet and digital technologies, smartphones have become an indispensable part of everyday life worldwide. According to the International Telecommunication Union (International Telecommunication Union, 2025), 82% of individuals aged 10 years or older worldwide own a mobile phone. Similarly, in Türkiye, Internet use among individuals aged 16–74 reached 90.9% in 2025 (Turkish Statistical Institute [TurkStat], 2025), reflecting the widespread integration of digital technologies into everyday life. These figures indicate that digitalization in Türkiye continues to progress in parallel with global trends, with smartphones playing an increasingly central role in shaping everyday behaviors. As multifunctional devices, smartphones facilitate communication, education, work, entertainment, information access, and online shopping by providing continuous, portable, and convenient access to digital services (Zhu et al., 2025). However, their constant availability and multifunctionality may also encourage excessive engagement, increasing the risk of problematic smartphone use (PSU) and associated functional impairments. Consequently, PSU has emerged as an important public health concern, particularly among university students, because of its adverse effects on mental health, academic performance, and face-to-face social interactions (Chen et al., 2025; Li et al., 2023).

Problematic smartphone use (PSU) should be distinguished from smartphone addiction. PSU is generally used as a non-diagnostic term to describe maladaptive or dysregulated patterns of smartphone use associated with negative consequences or interference in daily functioning, whereas the classification of smartphone-related problems as a behavioral addiction remains debated (Billieux et al., 2015a; Panova and Carbonell, 2018; Ryding and Kuss, 2020). Smartphone addiction is not currently recognized as an independent disorder in major psychiatric classification systems. Accordingly, the use of addiction-related theoretical models in the present study is intended to provide possible explanatory frameworks for psychological and behavioral processes associated with problematic use, rather than to imply that PSU is equivalent to an addictive disorder or that participants met diagnostic criteria for addiction. Throughout the manuscript, the term “addiction” is therefore retained only when referring to the terminology used in cited studies or to participants’ own subjective descriptions of their smartphone use.

A further distinction is necessary between problematic smartphone use (PSU) and problematic social media use (PSMU). Although these constructs are closely related, they are not interchangeable. PSMU refers specifically to dysregulated engagement with social media platforms, whereas PSU encompasses a broader range of smartphone-based activities, including social media use, messaging, gaming, video streaming, and other app-based behaviors. Recent evidence also suggests that PSU and PSMU may be associated with partly different psychological and motivational factors, further supporting their conceptual distinction despite their overlap (Giancola et al., 2025; Giancola et al., 2026).

### Prevalence and health correlates of problematic smartphone use

Having clarified these conceptual distinctions, it is important to consider the extent to which problematic patterns of smartphone use are reported among young adults and the health-related correlates associated with such use. Studies employing different conceptualizations and assessment approaches have reported substantial levels of problematic smartphone-related behaviors in young adult populations. For example, a UK study using an addiction-oriented measure reported elevated scores among 39% of young adults (Sohn et al., 2021), whereas the prevalence of PSU was reported as 33.8% among Spanish university students (Ramón-Arbués et al., 2025). Studies from China have likewise documented high and increasing levels of problematic smartphone-related behaviors among university students (Lyu et al., 2024; Mei et al., 2023). Recent research from Türkiye has also highlighted the relevance of psychological factors associated with problematic smartphone-related behaviors among adolescents and university students (Alkal, 2025; Akinci and Durmuş, 2025). Taken together, these findings indicate that problematic patterns of smartphone use represent an important concern among young adults and underscore the need to understand both their associated consequences and underlying psychological processes.

The PSU has been associated with a wide range of adverse physical, psychological, and social health outcomes. From a physical perspective, prolonged smartphone use is often characterized by a forward head posture and inadequate upper-limb support, resulting in increased static loading of the neck and shoulder muscles and a greater risk of musculoskeletal disorders (Gustafsson et al., 2017). In addition, PSU has been linked to several adverse lifestyle and physical health outcomes, including sedentary behavior (Yağci et al., 2024), unhealthy eating habits (Yılmaz et al., 2025), visual problems (Wang et al., 2020), reduced sleep duration, and increased daytime fatigue (Serim et al., 2026; Sohn et al., 2021). Beyond its physical consequences, PSU has consistently been associated with poorer mental health, including higher levels of depression and loneliness and lower subjective well-being (Elhai et al., 2019; Guo et al., 2020; Shao et al., 2024). Furthermore, intensive or problematic smartphone use has been associated with poorer interpersonal functioning, including reduced quality of face-to-face interactions (Dwyer et al., 2018), weakening relationships with family members and close friends (Hawi and Samaha, 2017), lowering interaction quality (Brown et al., 2016), and decreasing relationship satisfaction among romantic partners (Wang et al., 2017).

### Developmental processes of PSU

Although PSU should not be equated with a formally recognized addictive disorder, several theoretical frameworks may offer useful perspectives for understanding the psychological and behavioral processes associated with its development and maintenance. In particular, process-based and addiction-related models may help explain mechanisms such as reinforcement, habitual responding, emotion regulation, and diminished perceived control. In the present study, these frameworks are therefore considered as explanatory perspectives rather than diagnostic accounts of PSU.

The process-based model conceptualizes behavioral addictions as the result of learning and behavioral control mechanisms rather than discrete symptom clusters. According to this model, smartphone use is initially motivated by adaptive goals such as communication, entertainment, information seeking, or coping with negative emotions (Billieux et al., 2015b; Perales et al., 2020). When smartphone use is perceived as rewarding or effective in alleviating negative emotions, it is reinforced through positive or negative reinforcement. Over time, repeated reinforcement and exposure to environmental cues contribute to increasingly automatic patterns of use (Perales et al., 2020). A complementary perspective is provided by the Interaction of Person–Affect–Cognition–Execution (I-PACE) model, which conceptualizes problematic technology use as a dynamic process involving interactions among individual predispositions, affective and cognitive responses, use-related expectancies, and self-regulatory processes (Brand et al., 2016, 2019). From this perspective, personal characteristics and emotional experiences may interact with cognitive responses and reinforcement processes in ways that contribute to repetitive patterns of technology use.

Uses and Gratifications Theory suggests that individuals use smartphones to maintain social relationships, acquire information, seek entertainment, express their identities, and fulfill emotional needs (Katz et al., 1973). Repeated satisfaction of these needs reinforces smartphone use and may gradually contribute to the development of habitual use (Wegmann and Brand, 2019). Similarly, Compensatory Internet Use Theory suggests that individuals engage with digital technologies to cope with stress, loneliness, anxiety, and other adverse life experiences (Kardefelt-Winther et al., 2017). Although smartphone use may provide short-term emotional relief, negative reinforcement can maintain this behavior, and unresolved underlying problems may lead individuals to adopt smartphone use as a habitual coping strategy.

Theoretical models further suggest that the development of PSU is heterogeneous rather than uniform. In this regard, Billieux et al. (2015b) Pathway Model proposes that PSU may emerge through distinct psychological pathways, including excessive reassurance seeking, impulsivity, and reward sensitivity, rather than through a single developmental process. Accordingly, different patterns of smartphone use are thought to reflect different underlying psychological mechanisms (Canale et al., 2021).

In summary, existing theoretical frameworks converge on the view that PSU cannot be explained by a single cause. Although these models differ in their conceptual perspectives, they consistently describe PSU as a multidimensional phenomenon that emerges through the dynamic interplay of psychological needs, emotional processes, cognitive evaluations, learning mechanisms, reward systems, and individual predispositions over time. These theoretical frameworks are largely supported by quantitative evidence examining associations among psychological variables and behavioral outcomes. While such studies have identified risk factors and psychological correlates of PSU, they provide only limited insight into how individuals experience smartphone use in their everyday lives, how psychological needs, emotions, cognitions, and environmental influences are subjectively interpreted, and how these processes interact throughout the development of problematic use. Indeed, James et al. (2023) argue that existing research has predominantly assessed smartphone use through objective behavioral indicators, such as screen time, frequency of use, and time spent on specific applications, while paying comparatively little attention to the psychological meanings that individuals attribute to their smartphone use. Therefore, a deeper understanding of problematic smartphone-related experiences requires examining these psychological processes within the context of individuals’ lived experiences and everyday lives.

### The present study

Building on this gap, the present study focuses on how university students with intensive smartphone use subjectively experience and make sense of their smartphone use in everyday life. Previous qualitative research has provided valuable insights into students’ attitudes toward smartphone use, perceived reasons for use, and its consequences (e.g., Yang et al., 2021); however, a more integrated understanding of how emotional, cognitive, social, and contextual processes are experienced and interconnected in daily life remains limited. Importantly, the present study does not assume that intensive smartphone use is equivalent to PSU or smartphone addiction.

A phenomenological approach was adopted because it enables an in-depth examination of how individuals interpret and assign meaning to their lived experiences rather than reducing these experiences to predetermined behavioral indicators or diagnostic categories (Giorgi, 2018; Smith et al., 2009). Using in-depth semi-structured interviews complemented by 14-day micro-journals, the study sought to explore the meanings, functions, perceived difficulties, and psychological processes that participants associated with their smartphone use. By providing a contextualized account of these experiences, the study may contribute to a more nuanced understanding of PSU and inform future prevention and intervention efforts.

Accordingly, the study addressed the following research questions:

*RQ1*. How do university students with intensive smartphone use experience and make sense of their smartphone use in everyday life?

*RQ2*. What emotional, cognitive, social, and contextual functions and meanings do participants attribute to their smartphone use?

*RQ3*. How do participants perceive and describe aspects of their smartphone use that they experience as difficult to regulate or otherwise problematic?

## Method

### Research design

This study employed a qualitative phenomenological design to obtain an in-depth understanding of university students’ smartphone use experiences. A phenomenological approach was considered appropriate because it enables the exploration of smartphone use in all its aspects. Data were collected through semi-structured, in-depth interviews and analyzed using thematic analysis.

### Participants

The study sample consisted of 28 university students enrolled at Gümüşhane University, including 22 females and 6 males, aged between 20 and 26 years. Participants were recruited using a combination of purposive and convenience sampling from different departments within the Faculty of Health Sciences, with variation in academic department considered during recruitment to enhance diversity within the sample. However, participants’ specific departmental affiliations were not systematically recorded in the study dataset.

The inclusion criteria were: (1) being a university student, (2) owning a smartphone, (3) reporting a daily smartphone use duration of at least 4 h, and (4) being able to understand and speak Turkish. The criterion of ≥4 h of daily smartphone use was used to recruit participants with relatively intensive smartphone use and was not intended as a diagnostic or screening threshold for PSU. Previous research has used various duration-based thresholds when examining intensive or potentially problematic smartphone use; however, no standardized screen-time cut-off has been established for identifying PSU, and high usage duration alone does not necessarily indicate problematic use (Andrews et al., 2015; Harris et al., 2020; Ryding and Kuss, 2020). Accordingly, the present study does not assume that all participants exhibited PSU. Rather, the sampling criterion was used to recruit individuals with sufficient day-to-day smartphone engagement to provide rich accounts of their smartphone-related experiences.

Participant recruitment continued until data saturation was reached. During the later interviews, participants’ accounts increasingly repeated previously identified experiences, and no substantively new codes or thematic content emerged; recruitment was therefore concluded after 28 interviews. In qualitative research, saturation is commonly understood as the point at which additional data no longer contribute substantially new information or themes (Guest et al., 2006). Sample adequacy was also considered in relation to the information power of the dataset. According to the information power framework, the adequacy of a qualitative sample depends on factors such as the specificity of the sample, the scope of the study aim, the quality of the interview dialog, the use of established theoretical perspectives, and the analytical strategy (Malterud et al., 2016). The present sample comprised a relatively specific group of university students with intensive smartphone use, and the 28 in-depth interviews generated detailed accounts directly relevant to the study aims. The interview data were further complemented by 14-day micro-journals, which provided additional contextual information regarding participants’ everyday smartphone experiences. Taken together, these features were considered to provide sufficient information power for an in-depth exploration of the phenomenon. All participants volunteered to participate in the study. Sociodemographic characteristics of the participants are presented in Table 1.

**Table 1:** Sociodemographic characteristics of participants.

Participant	Age	Gender	Average daily screen time	Most frequently used digital activity
P1	20	Female	4–6 h	Social media
P2	23	Female	7–9 h	Social media
P3	23	Female	4–6 h	Movies, TV series, TV, etc.
P4	23	Female	7–9 h	Movies, TV series, TV, etc.
P5	21	Female	4–6 h	Social media
P6	20	Male	4–6 h	Social media
P7	21	Female	4–6 h	Messaging applications
P8	20	Female	7–9 h	Social media
P9	21	Male	4–6 h	Social media
P10	22	Female	10 h+	Social media
P11	21	Female	4–6 h	Social media
P12	21	Female	4–6 h	Social media
P13	21	Female	10 h+	Social media
P14	26	Male	4–6 h	Social media
P15	21	Female	4–6 h	Social media
P16	21	Male	4–6 h	Movies, TV series, TV, etc.
P17	22	Male	7–9 h	Social media
P18	24	Female	10 h+	Movies, TV series, TV, etc.
P19	20	Female	4–6 h	Social media
P20	21	Female	10 h+	Messaging applications
P21	23	Female	4–6 h	Social media
P22	21	Female	7–9 h	Movies, TV series, TV, etc.
P23	21	Female	4–6 h	Messaging applications
P24	21	Female	7–9 h	Social media
P25	22	Female	7–9 h	Social media
P26	22	Male	4–6 h	Social media
P27	23	Female	10 h+	Social media
P28	23	Female	7–9 h	Social media

### Data collection tools and procedure

Data were collected using a semi-structured interview guide and a micro-journal developed by the research team. The interview guide consisted primarily of open-ended questions designed to explore participants’ everyday smartphone experiences. Prior to the main data collection, the interview guide was pilot tested with two university students who were not included in the final sample. The pilot interviews were used to assess the clarity, comprehensibility, and flow of the questions and to identify potentially leading wording. Based on the feedback obtained, the wording and structure of the interview guide were revised before its use with the 28 participants in the main study. The complete final semi-structured interview guide is provided as Supplementary Material S1.

The micro-journal was a researcher-developed solicited daily diary form designed to capture participants’ smartphone-use experiences in their everyday contexts. Participants were asked to complete the micro-journal once daily for 14 consecutive days, recording their smartphone-use experiences together with the accompanying emotions and thoughts. Qualitative diary methods are particularly suited to capturing time-sensitive, context-specific, first-person accounts of experiences as they occur over time and may reduce some of the limitations associated with retrospective recall (Unterhitzenberger and Lawrence, 2022). The use of diary data alongside interviews can also provide complementary perspectives on participants’ experiences and support methodological triangulation (Unterhitzenberger and Lawrence, 2022). Daily diary designs have also been used in recent research on problematic mobile phone use; for example, Xu et al. (2024) employed a 14-day daily diary design with university students to examine day-to-day psychological experiences associated with problematic mobile phone use. In the present study, the micro-journal was not intended as a standardized psychometric instrument; therefore, conventional psychometric indices of validity and reliability were not applicable. Rather, methodological rigor was supported through repeated daily recording, participant-level comparison of journal entries with the corresponding interview accounts, joint review of the journal data by both researchers, and transparent documentation of the procedures used to integrate the two data sources.

All interviews were conducted by the first author, a psychologist with a PhD in psychological counseling who had received formal training in interviewing techniques and had previous experience conducting qualitative research and in-depth interviews. To minimize potential power dynamics and social desirability bias, the first author had no prior hierarchical or dual relationship with the participants. Specifically, the interviewer did not attend the participants’ classes and held no evaluative or grading authority over them. These clear boundaries and lack of evaluative power helped establish an egalitarian rapport, ensuring that participants could share their experiences freely and authentically without fear of academic or personal repercussions.

Interviews were conducted individually in a face-to-face setting and lasted approximately 30–45 min. A semi-structured format was used to provide consistency across interviews while allowing participants sufficient flexibility to elaborate on their lived experiences. With participants’ informed consent, all interviews were audio-recorded and subsequently transcribed verbatim. During transcription, all identifying information was removed to protect participant anonymity.

In addition to the interviews, participants completed a micro-journal designed to capture their everyday smartphone-use experiences. They were asked to complete the micro-journal daily for 14 consecutive days, documenting their smartphone use together with accompanying emotions and thoughts. The interview data constituted the primary source of analysis, whereas the micro-journals were used as a complementary source of contextual information.

### Researcher reflexivity

The research team recognized that their professional backgrounds, prior assumptions, and theoretical orientations could influence the processes of data collection, coding, and interpretation. The first author is a psychologist with a PhD in psychological counseling, has received formal training in interviewing techniques, and has experience conducting qualitative research and in-depth interviews. The second author is a social worker with a PhD in social work and training in qualitative research methods and interviewing techniques. Both researchers work in higher education settings and have professional experience interacting with university students, providing familiarity with the broader context in which participants’ smartphone-use experiences occur. At the same time, the researchers remained aware that such contextual familiarity could shape their expectations and interpretations. Given the study’s focus on intensive smartphone use and experiences perceived as difficult to regulate or problematic, the researchers remained attentive to the possibility of interpreting such use through a problem-focused or addiction-oriented lens. To limit this potential influence, participants’ accounts were approached as subjective and contextually situated experiences rather than being fitted into predetermined diagnostic categories, and intensive smartphone use was not assumed, by itself, to indicate PSU or smartphone addiction.

Reflexivity was supported throughout the data collection and analysis process through repeated engagement with the original interview transcripts and the maintenance of research notes documenting coding and theme development. The codes, relevant data extracts, and emerging thematic structure were independently reviewed by the second researcher, and alternative interpretations were discussed between the researchers. When differences in interpretation arose, the researchers returned to the original participant accounts and reconsidered the scope, boundaries, and meanings of the relevant codes and themes. During data collection, open-ended questions and neutral follow-up probes were used to minimize unnecessary direction of participants’ responses toward the researchers’ expectations.

### Data analysis

The interview data were analyzed using thematic analysis, a flexible qualitative analytic method that enables the systematic identification, organization, and interpretation of patterns of meaning within a dataset (Braun and Clarke, 2006). This approach was considered appropriate because it facilitates an in-depth exploration of how university students experience and make sense of their smartphone use. Following transcription of the audio-recorded interviews, the data were imported into computer-assisted qualitative data analysis software to facilitate data management and organization. The software was used to organize coded text segments, compare related codes, and review the thematic structure. However, all analytical decisions regarding code development, theme generation, and interpretation were made by the research team.

The semi-structured interview data constituted the primary source of analysis. After the interview-derived thematic structure had been developed, the micro-journal data were analyzed as a complementary qualitative data source. Participants used participant codes that allowed each 14-day micro-journal to be matched with the corresponding interview account. The journal entries were coded and jointly reviewed by both researchers. At the participant level, the coded journal data were compared with the corresponding interview narratives and with the themes developed from the interview data. The comparison focused on identifying convergence, elaboration, divergence, and any additional information across the two data sources. This procedure was informed by qualitative diary approaches in which diary and interview data are combined through methodological triangulation (Unterhitzenberger and Lawrence, 2022).

The micro-journals largely converged with and elaborated the patterns identified in the interview data, particularly by providing day-to-day contextual detail regarding smartphone use, emotions, and thoughts. Although the researchers remained open to identifying new codes or themes arising from the journal data, no substantively new theme emerged that required modification of the interview-derived thematic structure. The micro-journals were therefore integrated into the overall interpretation as a corroborative and elaborative data source rather than being reported as a separate thematic dataset.

The thematic analysis of the interview data followed the six-phase framework proposed by Braun and Clarke (2006). First, the researchers familiarized themselves with the data by repeatedly reading the interview transcripts. Second, meaningful segments of text relevant to the research objectives were systematically coded to generate initial codes. Third, similar codes were grouped to develop preliminary themes. Fourth, the candidate themes were reviewed and refined by comparing them with both the coded extracts and the complete dataset to ensure internal coherence and clear distinctions between themes. Fifth, the themes were defined, refined, and named to accurately reflect their underlying meanings. Finally, the themes were reported and illustrated with representative quotations from the participants to support the interpretation of the findings (Braun and Clarke, 2006).

The reporting of the present study was reviewed with reference to the Consolidated Criteria for Reporting Qualitative Research (COREQ) checklist to enhance transparency regarding the research team, study design, data collection, analysis, and reporting of findings (Tong et al., 2007).

### Coding process, analytical consistency, and credibility

The coding process was primarily conducted by the first researcher. All interview transcripts were read repeatedly to achieve familiarity with the data, after which meaningful data segments were systematically coded in accordance with the study objectives, resulting in the development of initial codes. Following the initial coding, a second researcher independently reviewed the codes, the corresponding data extracts, and the thematic structure. The purpose of involving a second researcher was to assess the extent to which the codes and themes accurately reflected the data, evaluate the clarity of thematic boundaries, and ensure the analytical consistency of the interpretations.

To enhance the credibility and analytical rigor of the study, data collection and analysis were conducted systematically. Interviews with university students were audio-recorded, transcribed, and repeatedly reviewed during the analysis process to compare coding and theme development with the original data. Research notes on the coding and theme development process were maintained, and the representativeness of the themes was regularly reviewed. To enhance the credibility of the findings, each theme was supported by direct quotations from the participants. To strengthen analytical consistency, the coding and thematic structure were reviewed by a second researcher. Differences in interpretation were discussed among the researchers, and the scope, boundaries, and conceptual meaning of the themes were re-evaluated.

To enhance transparency regarding the analytical process, Table 2 provides illustrative examples of how participant data extracts were coded and subsequently organized into subthemes and overarching themes.

**Table 2:** Illustrative examples of the coding process from participant quotations to subthemes and themes.

Illustrative quotation	Code	Subtheme	Theme
“Honestly, it’s mostly because I’m bored. If I had something else to do, I would not want to spend so much time on these things.” (P2, Female, 23 years)	Boredom	Mood during Smartphone Use	Relationship with the Smartphone and Perceived Control
“When I’m stressed or feeling down, I turn to my smartphone to get away from it all. It keeps me distracted, but it only works in the moment.” (P12, Female, 21 years)	Temporary escape from problems	Cognitive Escape	Coping Functions of Smartphone Use
“At night, I mostly come across more depressing content, while during the day I see more funny posts. It also seems to change depending on my mood.” (P3, Female, 23 years)	Content changes according to mood	Content Algorithm	Content and Social Feedback
“Comparing myself to others makes me feel more stressed and inadequate.” (P2, Female, 23 years)	Feeling inadequate	Negative Self-Evaluation	Self-Evaluation
“After a while, my eyes start to hurt.” (P6, Male, 20 years)	Eye strain	Physical Effects	Daily Use Patterns and Effects

### Ethical considerations

Ethical approval for the study was obtained from the Gümüşhane University Scientific Research and Publication Ethics Committee (Decision no. E-76551937-108.99-413605, dated 25/03/26). Before the interviews, participants were informed about the purpose of the study, the voluntary nature of participation, their right to withdraw from the study at any time, and the confidentiality of the data. Written informed consent was obtained from all participants prior to data collection.

## Results

The interview data were analyzed using thematic analysis with the support of computer-assisted qualitative data analysis software. Analysis of the interviews with 28 university students (P1–P28) generated five overarching themes: (1) relationship with the smartphone and perceived control, (2) coping functions of smartphone use, (3) content and social feedback, (4) self-evaluation, and (5) daily use patterns and effects. Each theme is described below together with its corresponding subthemes and supported by representative participant quotations. Figure 1 presents a conceptual framework summarizing the thematic structure identified in the data.

**Figure 1:**
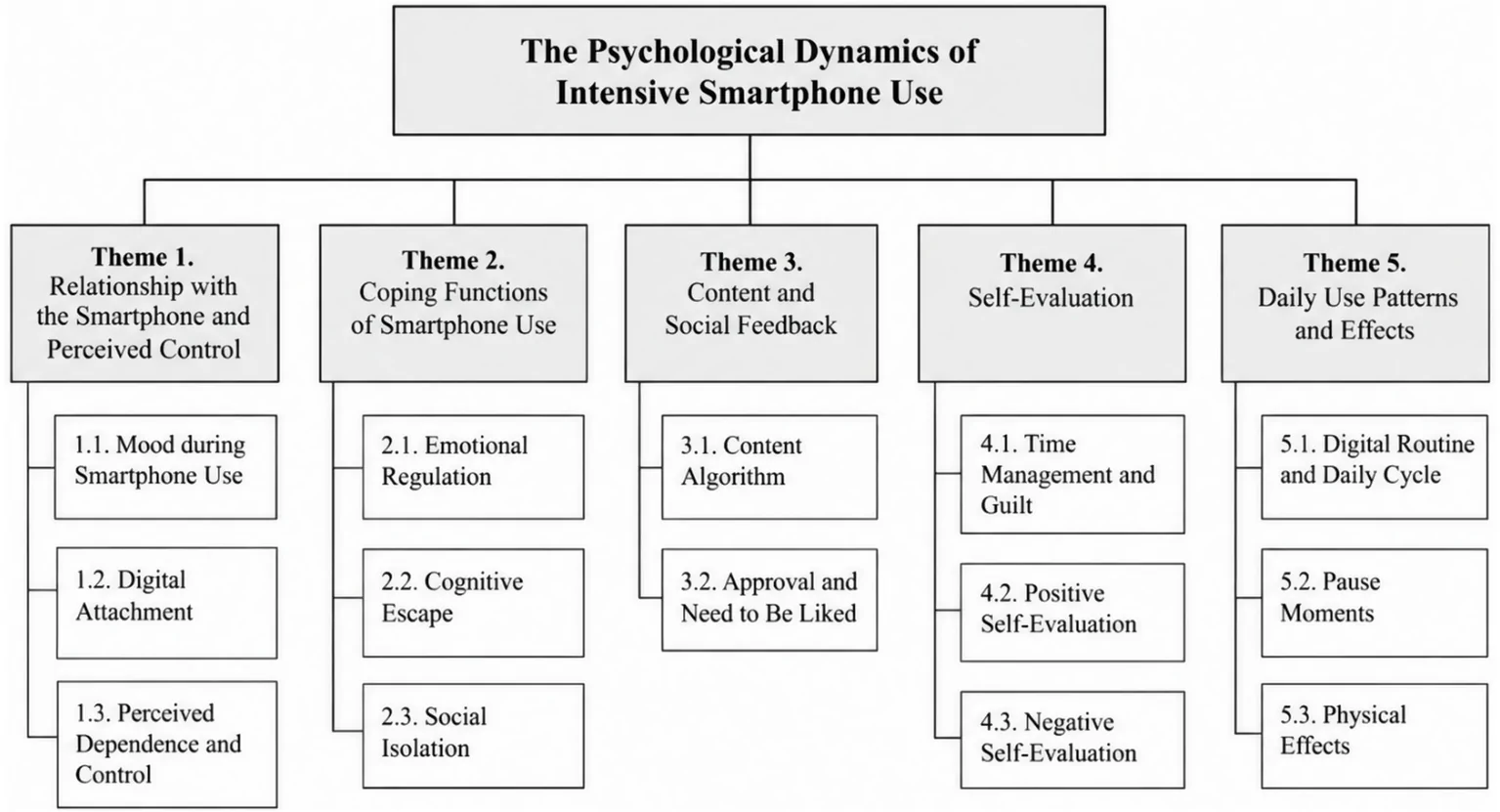
Thematic structure of the psychological dynamics of intensive smartphone use. The framework illustrates the five major themes and related subthemes identified from participants’ lived experiences through thematic analysis. It is intended to provide a conceptual summary of the findings rather than to represent causal relationships.

### Theme 1: relationship with the smartphone and perceived control

The findings revealed that, for most participants, smartphone use intensified in response to changes in emotional state. Some participants also described the smartphone not only as a functional device but as a source of comfort when available and a source of emptiness when absent. Experiences related to dependency and perceptions of control emerged as prominent aspects of this theme.

#### Mood during smartphone use

Participants reported that the duration and intensity of smartphone use were closely related to their emotional experiences, with usage increasing particularly during periods of negative mood. Their narratives indicate that changes in emotional state were among the most important factors shaping smartphone use.

*“Honestly, it’s mostly because I’m bored. If I had something else to do, I wouldn’t want to spend so much time on these things. I’d actually rather be doing other activities.”* (P2, Female, 23 years).

*“When I’m feeling down or overthinking something, I use it as a way to escape from my own thoughts.”* (P13, Female, 21 years).

*“I tend to use it more when I’m feeling sad, having problems in my relationships, or when I just want to be alone.”* (P22, Female, 21 years).

Participants’ narratives suggest that increased smartphone use was not associated with a single emotional state but occurred across a range of negative experiences, including sadness, loneliness, boredom, feelings of worthlessness, and persistent negative thoughts. These findings indicate that diverse negative emotional experiences may constitute a common experiential context that encourages participants to engage in smartphone use.

#### Digital attachment

Participants described the smartphone not only as a technological device for communication but also as an object that held diverse emotional meanings in their lives. Illustrative quotations from the participants are presented below:

*“I spend most of my time on my phone. Whenever it’s not with me, I feel insecure.”* (P7, Female, 21 years).

*“When I don’t have my smartphone with me, I feel like something is missing and I get really restless. It feels like I’ve lost something really important.”* (P19, Female, 20 years).

*“I feel like my smartphone is the only thing that really understands me.”* (P15, Female, 21 years).

*“Our phones have almost become like a sixth sense. When I don’t have mine with me, I feel like something is missing, and like I’m falling behind something.”* (P26, Male, 22 years).

Participants’ narratives indicate that smartphone use is experienced as a strong psychological attachment that extends beyond its functional purposes. The absence of the smartphone was associated with feelings of emptiness, unease, and insecurity, whereas its presence was described as providing a sense of security and comfort.

#### Perceived dependence and control

Participants’ narratives further indicated that their evaluations of smartphone use were shaped by subjective perceptions of dependence and control. Some participants explicitly described themselves as “addicted”; however, these expressions are presented here as participants’ own interpretations of their smartphone use rather than as evidence of a clinically assessed addictive disorder. Illustrative quotations are presented below.

*“These days, I think we’re all addicted to our phones. I know I am, but I kind of pretend I’m not. I guess we all do because it’s easier that way.”* (P1, Female, 20 years).

*“I’d say I’m addicted because my smartphone takes up a huge part of my daily life.”* (P21, Female, 23 years).

*“To be honest, I am addicted to my smartphone because I’m constantly worried about keeping it charged.”* (P28, Female, 23 years).

Participants described smartphone use as occupying a substantial role in their daily lives and reported difficulties in limiting their use. These accounts reflect subjective experiences of dependence and reduced perceived control. However, because no clinical or diagnostic assessment of smartphone addiction was conducted, participants’ self-descriptions should not be interpreted as evidence of an addictive disorder.

### Theme 2: coping functions of smartphone use

This theme focuses on how participants experience smartphone use in response to stress, anxiety, loneliness, and persistent negative thoughts. The narratives indicate that smartphone use served functions related to relaxation, distraction, escaping intrusive thoughts, and seeking solitude during these experiences.

#### Emotional regulation

Participants described smartphone use not only as a way to pass the time but also as a coping strategy that they relied on to manage negative emotions, calm themselves, relax, and reduce their emotional burden.

*“My smartphone helps me when I’m stressed. It helps me relax, gives me hope again, and helps me cope with stress.”* (P14, Male, 26 years).

*“It mostly helps me relax because the funny things I see on social media make me feel better.”* (P15, Female, 21 years).

*“Listening to music on my smartphone calms me down and helps me let go of my stress.”* (P23, Female, 21 years).

Participants’ shared experiences suggest that smartphone use was perceived as a means of alleviating emotional burden and providing temporary relief. This sense of relief was most commonly associated with listening to music, watching videos, and engaging with entertainment content.

#### Cognitive escape

Participants also reported turning to their smartphones when they were preoccupied with persistent thoughts or faced problems they found difficult to resolve. Their narratives indicate that smartphone use was experienced as a way of escaping intrusive thoughts and redirecting attention.

*“It just keeps the voice in my head occupied. I’m really just pushing my thoughts aside for a while.”* (P5, Female, 21 years).

*“When I’m stressed or feeling down, I turn to my smartphone to get away from it all. It keeps me distracted, but it only works in the moment.”* (P12, Female, 21 years).

Participants’ shared experiences indicated that smartphone use was perceived by some participants as a way of escaping intense thoughts. However, several participants also emphasized that this effect was only temporary.

#### Social isolation

Participants described the impact of smartphone use on their social relationships in different ways. Illustrative quotations from the participants are presented below.

*“Even when I’m out at a café with my closest friends, instead of talking to each other, we just end up on our phones. It’s like we’re together, but there’s no real interaction.”* (P22, Female, 21 years).

*“One day, when I didn’t want to talk to anyone and just wanted to be alone, I shut myself off from everyone and spent the whole day on my phone.”* (P27, Female, 23 years).

Participants’ narratives indicated that smartphone use was experienced by some participants as limiting face-to-face interactions, whereas others described it as accompanying or reinforcing their desire to be alone.

### Theme 3: content and social feedback

This theme includes participants’ experiences of interacting with social media content. The narratives indicate that participants generally interpreted the content they encountered in relation to their own emotions, interests, and everyday experiences. Some participants also described social media posts and the reactions to those posts as becoming part of their personal experiences.

#### Content algorithm

Participants reported that the content appearing in their social media feeds was not perceived as random but rather as reflecting their interests, emotional states, or daily experiences. Illustrative quotations from the participants are presented below.

*“At night, I mostly come across more depressing content, while during the day I see more funny posts. It also seems to change depending on my mood.”* (P3, Female, 23 years).

*“The content I see is based on the words and conversations we’ve had throughout the day. So, it kind of feels like a replay of my day.”* (P9, Male, 21 years).

*“When I watch funny videos, I keep getting more funny posts.”* (P24, Female, 21 years).

Participants’ narratives indicated that social media content was experienced in relation to their personal interests, previous interactions, and current emotional states. Participants described their social media feeds as reflecting and extending their everyday experiences.

#### Approval and need to be liked

Some participants emphasized the importance of receiving approval and positive feedback from their social circles through smartphone use. Illustrative quotations from the participants are presented below.

*“Whenever I’m feeling beautiful, the first thing I do is take a picture and post it.”* (P5, Female, 21 years).

*“When I post a photo of myself on social media and get a lot of likes, it really makes my day.”* (P12, Female, 21 years).

*“When I’m happy, social media is where I want to share that moment.”* (P13, Female, 21 years).

Participants’ narratives indicated that, for some participants, receiving likes on social media posts was associated not only with increased visibility but also with feeling better about themselves. Similarly, posting on social media was most often associated with periods of positive emotions. These shared experiences suggest that posting content and receiving likes and feedback from one’s social circle constituted an important aspect of the social media experience for some participants.

### Theme 4: self-evaluation

This theme encompasses participants’ self-evaluations associated with intensive smartphone use and, where relevant, with social media experiences. While time-management concerns were generally related to smartphone use, social comparison and related self-evaluations were described primarily in the context of social media. The narratives indicate that smartphone use was associated with different forms of self-evaluation related to time management, self-perception, and social comparison.

#### Time management and guilt

Participants reported that, following intensive smartphone use, they felt they had not used their time efficiently, which often led to feelings of guilt.

*“When I spend too much time on my phone, I feel guilty because I know I’m wasting my time.”* (P3, Female, 23 years).

*“When I notice my screen time going up, I feel guilty because I’m just scrolling through pointless Reels or wasting time on things that don’t really matter.”* (P5, Female, 21 years).

*“Knowing I could be doing something more meaningful for myself instead of being glued to my smartphone makes me feel guilty.”* (P11, Female, 21 years).

Participants’ narratives indicated that smartphone use was predominantly perceived as a source of wasted time and inefficiency. For many participants, this experience was accompanied by feelings of guilt and regret.

#### Positive self-evaluation

Some participants reported comparing themselves with others through the content they encountered on social media and described how this process influenced their self-evaluations.

*“While I’m scrolling through Reels, I catch myself thinking, “I could do even better than that.” Honestly, it gives me a confidence boost.”* (P12, Female, 21 years).

*“Some of the content I see makes me feel like I should believe in myself more and recognize my own potential. If they can do it, then I can do it too.”* (P19, Female, 20 years).

Participants’ shared experiences indicated that some participants associated the content they encountered on social media with positive self-evaluations. Their narratives suggest that the digital experience extended beyond content consumption and was linked to self-confidence, recognizing one’s potential, and personal growth.

#### Negative self- evaluation

Participants’ statements also revealed that social comparison on social media did not always result in positive self-evaluations. For some participants, exposure to idealized or ostentatious lifestyles on social media was associated with negative emotions.

*“Comparing myself to others makes me feel more stressed and inadequate.”* (P2, Female, 23 years).

*“What I see makes me feel like I don’t spend enough time on myself and that I cannot travel or enjoy life as much as other people do.”* (P6, Male, 20 years).

*“I start to think, “What am I doing wrong?” These comparisons pull me down even more and drain the energy I have inside me.”* (P13, Female, 21 years).

*“I notice the places people visit and the clothes they wear. Then I start thinking, “Why can’t I do those things? Why can’t I dress like that?”.”* (P24, Female, 21 years).

Participants’ narratives indicated that social comparisons based on social media content were often accompanied by feelings of inadequacy. In particular, posts depicting the lives of others were associated with negative self-evaluations among participants.

### Theme 5: daily use patterns and effects

This theme encompasses the patterns of smartphone use within participants’ daily lives. The narratives indicate that smartphone use follows recurring routines at specific times of the day, while these routines may be interrupted during certain experiences. Participants also reported experiencing physical effects associated with intensive smartphone use. A word cloud of the most frequently used words in participants’ narratives is presented in Figure 2.

**Figure 2:**
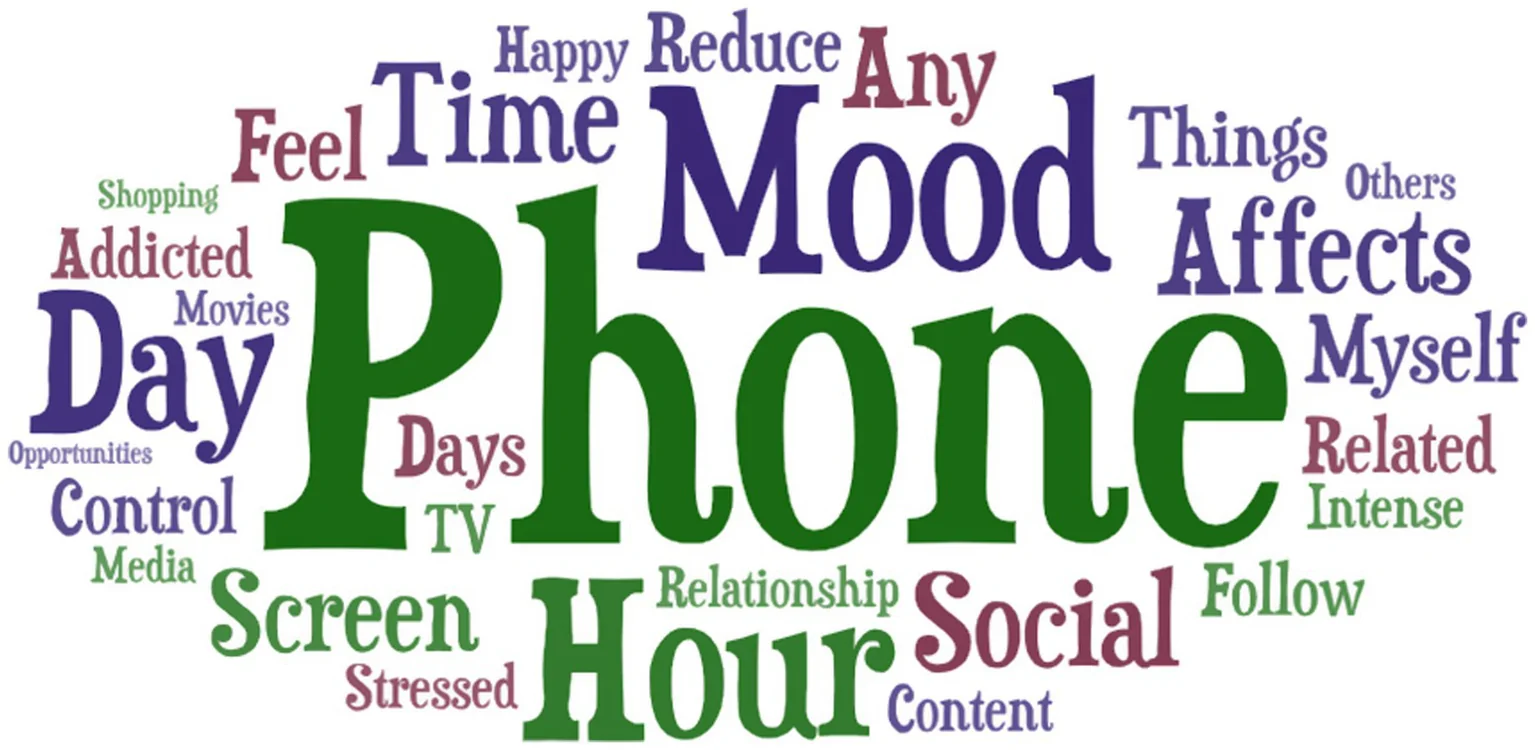
Word cloud of the most frequently used words in participants’ narratives.

#### Digital routine and daily cycle

Participants reported that smartphone use had become integrated into their daily routines from the first moments of the day. Their narratives indicate that reaching for the smartphone immediately after waking up was experienced by many participants as one of their initial daily behaviors.

*“The first thing I do when I wake up is check my phone.”* (P2, Female, 23 years).

*“As soon as I open my eyes, I check my messages and calls.”* (P6, Male, 20 years).

*“The moment I wake up, I reach for my phone.”* (P13, Female, 21 years).

Participants’ shared experiences indicated that smartphone use had become a regular daily habit, particularly at the beginning of the day.

#### Pause moments

Participants reported that certain experiences resulted in noticeable changes in their smartphone use. According to their narratives, smartphone use decreased particularly during periods when social relationships were prioritized.

*“When I’m out in nature and getting some fresh air, my smartphone doesn’t even cross my mind. I just let myself enjoy being there.”* (P18, Female, 24 years).

*“When I’m with my boyfriend, I use my smartphone much less. Sometimes I don’t even pick it up at all.”* (P28, Female, 23 years).

*“On the days I’m working, I’m usually active and social. I don’t really have the time or the need to check my phone.”* (P21, Female, 23 years).

According to participants’ statements, social relationships, time spent in nature, and daily responsibilities emerged as factors associated with reduced smartphone use. These findings suggest that smartphone use varies according to the context in which individuals are engaged.

#### Physical effects

Some participants reported experiencing various physical discomforts following intensive smartphone use. Illustrative quotations from the participants are presented below.

*“I usually notice that my neck starts to hurt after I’ve been on my smartphone for a while.”* (P1, Female, 20 years).

*“After a while, my eyes start to hurt.”* (P6, Male, 20 years).

*“It feels like my head is going to explode, and I find myself completely disconnected from life.”* (P13, Female, 21 years).

Participants reported experiencing various physical discomforts, such as headaches and eye strain, following intensive smartphone use. These narratives suggest that smartphone use may have physical as well as psychological consequences.

Taken together, the five themes illustrate how intensive smartphone use was experienced across interconnected emotional, cognitive, social, and behavioral dimensions. Participants’ accounts linked smartphone use with emotional states and perceived attachment and control, coping and cognitive escape, interactions with digital content and social feedback, self-evaluative experiences, and recurring patterns of everyday use. These findings suggest that participants experienced these dimensions as interconnected aspects of their everyday smartphone use rather than as isolated experiences.

## Discussion

This study explored university students’ experiences of intensive smartphone use, with particular attention to the psychological processes and experiences that participants perceived as difficult to regulate or problematic. By focusing on participants’ lived experiences, the study provides a qualitative perspective on how smartphone use is intertwined with emotional, cognitive, social, and behavioral processes in everyday life. In this respect, the findings may contribute to a more nuanced understanding of problematic smartphone use beyond usage duration and other behavioral indicators.

### Discussion of findings regarding the theme of relationship with the smartphone and perceived control

The first theme identified in this study was relationship with the smartphone and perceived control. This theme highlights that university students’ smartphone use is primarily associated with negative emotional experiences. Participants reported turning to their smartphones in response to various emotional states, including stress, loneliness, boredom, sadness, and intense thoughts, and described smartphones as accessible tools that help them regulate and cope with these experiences. Consistent with Compensatory Internet Use Theory, individuals may engage with digital environments as a means of alleviating psychological distress and coping with adverse life experiences (Kardefelt-Winther, 2014). Previous empirical research similarly indicates that smartphone use is closely intertwined with individuals’ psychological states and may function as a coping strategy in response to negative emotions (Hoffner and Lee, 2015; Shahidin et al., 2022). These findings suggest that smartphone use is not only driven by the inherent characteristics of the technology itself; rather, it is shaped by individuals’ emotional needs and psychological conditions.

Another finding of the first theme is that participants’ relationship with their smartphones was sometimes experienced as a strong psychological attachment. Participants frequently associated the absence of their smartphones with feelings of emptiness, unease, and insecurity, and some explicitly described themselves as being “addicted” to their smartphones. Importantly, these statements represent participants’ subjective perceptions of dependence and reduced control rather than evidence of a clinically established addictive disorder. This distinction is particularly relevant because experiences such as excessive use, perceived loss of control, and negative consequences may resemble features commonly associated with addiction but do not, by themselves, establish the presence of an addictive disorder (Panova and Carbonell, 2018). These subjective experiences may be interpreted in light of the Interaction of Person–Affect–Cognition–Execution (I-PACE) model (Brand et al., 2016, 2019), which proposes that affective and cognitive responses, reinforcement processes, and executive control may interact in the development of repetitive technology-related behaviors. In the context of the present findings, the I-PACE model provides a useful theoretical perspective for understanding participants’ perceived difficulty in regulating smartphone use; however, the present qualitative data do not provide evidence of an addictive disorder or of changes in executive functioning.

### Discussion of findings regarding the theme of coping functions of smartphone use

The first finding of the second theme is that participants experienced their smartphones as a means of regulating negative emotions such as stress and anxiety. This suggests that smartphone use may serve as an emotion regulation strategy for managing psychological distress. According to emotion regulation theory, individuals employ various cognitive and behavioral strategies to reduce the intensity of negative emotions and restore emotional balance (Gross, 2015). Within this framework, smartphone use can be understood as a readily accessible resource that provides temporary emotional relief. Consistent with this interpretation, Shahidin et al. (2022) reported a significant association between difficulties in emotion regulation and PSU. Similarly, studies examining motivations for smartphone use have shown that individuals frequently use their smartphones to relax, reduce stress, and regulate their emotions (Hoffner et al., 2016; Mostyn Sullivan and George, 2023). Taken together, the present findings suggest that, rather than only filling leisure time, smartphone use functions as an accessible coping resource through which students manage negative emotions and temporarily restore emotional equilibrium.

Another important finding of the second theme is that smartphone use also served as a form of cognitive escape, providing participants with temporary relief from persistent thoughts and mental burden. This suggests that, beyond regulating emotions, smartphone use may also function to reduce cognitive distress by redirecting attention away from stressors. According to emotion regulation theory, attentional deployment involves shifting attention away from negative stimuli and can provide short-term psychological relief (Gross, 2015). Consistent with this perspective, Elhai et al. (2018) found that rumination plays a significant role in the association between depression, social anxiety, and PSU, suggesting that individuals may use their smartphones to disengage from repetitive negative thoughts. Participants consistently reported that this relief was only temporary and did not resolve the underlying source of distress. This finding is consistent with coping theory, which proposes that avoidance-oriented coping strategies may provide immediate psychological relief but fail to address the source of stress, thereby increasing the likelihood that the same coping behavior will be repeated in the future (Lazarus and Folkman, 1984). The present findings suggest that smartphone use also functions as a form of cognitive escape by providing a temporary distraction from distressing thoughts and unresolved problems. Participants’ accounts of temporary relief are consistent with theoretical perspectives proposing that short-term relief may be associated with repeated use. However, the present findings cannot establish whether temporary relief causally reinforces subsequent smartphone use. Similar experiences were reported by Yang et al. (2021), who identified escapism as a perceived reason for problematic smartphone use among university students. Some participants in their study also described using smartphones to withdraw from or avoid face-to-face social interaction. These findings closely parallel the present participants’ accounts of both cognitive escape and reduced interpersonal engagement.

The final noteworthy finding within the second theme is that, for some participants, smartphone use gradually came to replace face-to-face social interaction. According to the Displacement Hypothesis, increased engagement in digital environments may reduce direct interactions with family and friends, thereby diminishing the quality of interpersonal relationships (Kraut et al., 1998). Consistent with this perspective, Przybylski and Weinstein (2013) demonstrated that the mere presence of mobile phones during interpersonal interactions can reduce both the quality of face-to-face conversations and perceptions of interpersonal closeness. Similarly, Misra et al. (2016) found that the presence of smartphones during conversations is associated with lower levels of empathy and communication satisfaction. Participants’ accounts indicated that intensive smartphone use and reduced face-to-face interaction were sometimes experienced together. This pattern is consistent with previous research reporting associations between smartphone or social media use and social isolation, loneliness, and poorer social functioning (Liu Y. et al., 2026; Liu T. et al., 2026; Sreehari et al., 2025). However, the present phenomenological data do not allow conclusions regarding the direction or temporal ordering of these relationships. Accordingly, the findings are interpreted as reflecting a perceived association between smartphone use and reduced face-to-face interaction rather than evidence that smartphone use causes or maintains social withdrawal.

### Discussion of findings regarding the theme of content and social feedback

Before interpreting the findings of this theme, it is important to distinguish smartphone use from social media use. Smartphones are multifunctional devices that support a wide range of activities, whereas problematic social media use (PSMU) refers specifically to dysregulated engagement with social media platforms. Although PSU and PSMU are closely related, they should not be treated as interchangeable constructs, as their psychological and motivational correlates may differ (Giancola et al., 2025, 2026). This distinction is particularly relevant to the present findings because social media was the most frequently used smartphone activity for a substantial proportion of participants, and several experiences within the Content and Social Feedback theme, including algorithmic personalization, likes, social feedback, and social comparison, were specifically described in the context of social media. These findings should therefore be interpreted as social-media-related processes occurring within participants’ broader smartphone-use experiences rather than as mechanisms that necessarily characterize all forms of smartphone use.

The first notable finding within the third theme is that participants perceived the social media content stream not as a random sequence of information, but as a dynamic and personalized system shaped by their emotions, interests, and previous digital interactions. This finding suggests that participants viewed digital content not only as a passive media environment for consumption, but as an interactive ecosystem that continuously adapts to and responds to their behavior. This interpretation is consistent with the concept of Perceived Algorithmic Sensitivity proposed by Taylor and Chen (2024), which suggests that users perceive personalized algorithmic recommendations as signals that the platform understands, recognizes, and validates their preferences and identity. Similarly, Oh et al. (2026) argued that users no longer perceive algorithms as passive technological tools; rather, by understanding their underlying logic and developing strategies to influence them, they engage with algorithms as interactive systems. Likewise, recent studies have shown that recommendation algorithms, particularly on platforms such as TikTok, are highly responsive to user behavior and rapidly adapt in ways that shape the user experience (Mosnar et al., 2025; Kang et al., 2026). A noteworthy finding of the present study is that participants emphasized their subjective experience of the algorithm rather than its technical functioning. For the participants, the content feed was perceived not only as a technology-driven recommendation system but as a digital environment that reflected their moods, daily experiences, and personal interests.

Another finding within the third theme is that, for some participants, likes and feedback received through social media interactions were experienced not only as forms of digital engagement but also as sources of social validation that influenced their emotional states. The need for acceptance and positive evaluation from others has long been recognized as a fundamental psychological need (Crowne and Marlowe, 1964; Leary and Allen, 2011). Within this framework, likes and other forms of feedback on social media can be understood as digital expressions of social validation. Consistent with this perspective, Sherman et al. (2016) demonstrated that receiving likes on social media activates brain regions associated with reward processing, particularly among young people, suggesting that online social feedback can produce psychologically rewarding experiences. Similarly, Ariel and Nir (2025) argued that visibility and like indicators on digital platforms have become important social cues that shape individuals’ perceptions of success and self-worth. Consistent with this perspective, some participants in the present study described posting content and receiving feedback as an extension of sharing their positive emotions and maintaining visible connections with their social environment. In particular, participants’ statements indicating that the number of likes influenced their daily mood suggest that social media feedback may become integrated into individuals’ self-evaluation processes.

### Discussion of findings regarding the theme of self-evaluation

The first notable finding within the fourth theme is that intensive smartphone use was perceived by participants not only as a waste of time but also as an experience associated with negative self-evaluations. Participants frequently described their smartphone use with expressions such as “wasted time” and “being unproductive,” and reported that these evaluations were accompanied by feelings of guilt. This finding is consistent with previous literature. For example, Halfmann et al. (2024) found that individuals experience guilt when smartphone use conflicts with personally important goals, such as studying or completing attention-demanding tasks. Similarly, Lu et al. (2023) demonstrated that screen-time feedback can increase cognitive dissonance when it conflicts with individuals’ perceptions of their own smartphone use, thereby eliciting feelings of guilt. Likewise, Zwanenburg and Tran (2022) conceptualized the continued use of smartphones despite recognizing that one should stop as Conflicted Technology Use, suggesting that this conflict is associated with self-evaluative emotions such as guilt and regret. Overall, the present finding is consistent with the literature indicating that guilt may arise when individuals perceive a discrepancy between their intended and actual smartphone use.

The findings related to the second and third subthemes of the fourth theme suggest that social comparisons in social media environments do not produce uniform psychological outcomes among university students. Participants’ narratives indicated that, for some individuals, comparing themselves with others promoted self-confidence and motivation for personal growth, whereas for others, the same process was associated with feelings of inadequacy, worthlessness, and negative self-evaluation. This finding is consistent with Social Comparison Theory by Festinger (1954), which proposes that individuals compare themselves with others to evaluate their own abilities, achievements, and personal characteristics. However, subsequent research has demonstrated that the psychological consequences of social comparison are not uniform. Upward social comparisons on social media have been associated with feelings of inadequacy and lower self-esteem in some individuals (Taylor and Armes, 2024; Kavaklı and Ünal, 2021), whereas in other cases they have been linked to greater inspiration, motivation, and self-improvement when the achievements of the comparison target are perceived as attainable (Noon and Meier, 2019; Peng et al., 2019). In this process, psychological mechanisms such as benign envy and motivation for self-improvement may influence individuals’ responses to social comparison. Consequently, the psychological effects of social comparison may vary according to the direction of the comparison, the context in which it occurs, and the individual’s interpretation of the comparison.

### Discussion of findings regarding the theme of daily use patterns and effects

The findings indicate that, for many participants, smartphone checking was experienced as a highly routinized and sometimes seemingly automatic part of everyday life. Participants frequently described checking their smartphones immediately after waking and during available moments throughout the day. These accounts resemble characteristics of habitual behavior described in the broader habit literature (Wood and Neal, 2007; Lally et al., 2010). However, because the present study did not prospectively assess habit formation, the findings should not be interpreted as demonstrating that repeated smartphone use progressively developed into habitual behavior. Rather, participants’ narratives indicate that their current patterns of use were subjectively experienced as routine and, at times, automatic. This interpretation is also broadly compatible with theoretical models such as I-PACE, which propose that repeated technology use may become increasingly habitual over time (Brand et al., 2016, 2019), although this developmental mechanism cannot be established from the present data.

However, the findings also suggest that smartphone use is not a completely rigid or unchangeable behavioral pattern. Rather, participants’ experiences indicate that smartphone use may be shaped not only by individual tendencies but also by the social and physical contexts in which it occurs. This observation is consistent with research on habitual behavior, which suggests that changes in environmental context can disrupt automatic behavioral patterns and facilitate the adoption of alternative behaviors (Wood and Neal, 2007; Verplanken and Wood, 2006). For example, spending time in nature or being involved in a romantic relationship may place individuals in contexts that differ from their usual digitally oriented environments. Such contextual changes may reduce exposure to cues (e.g., idleness or boredom) that typically trigger automatic smartphone checking, thereby promoting more intentional patterns of smartphone use. Consistent with this perspective, major life events and contextual changes can provide critical opportunities for the restructuring of habitual behaviors (Walker et al., 2015). Accordingly, the present findings indicate that engagement in offline experiences, such as meaningful social relationships, contact with nature, and goal-directed daily activities, may serve as contextual factors associated with reduced smartphone use.

The final finding within this theme is that participants reported a range of physical complaints associated with intensive smartphone use. Previous research has increasingly documented the adverse effects of PSU on headaches, eye strain, musculoskeletal problems, sleep disturbances, and overall physical health (Alotaibi et al., 2022; Zhu et al., 2025). However, a notable finding of the present study is that participants reported continuing to use their smartphones despite being aware of these physical symptoms. This suggests that awareness of physical discomfort alone may not necessarily lead participants to reduce their smartphone use. As reflected across the previous themes, participants’ experiences indicate that smartphone use is closely linked to multiple psychological and behavioral processes, including emotion regulation, cognitive escape, social comparison, and habitual use. Taken together, these findings illustrate the multifaceted emotional, cognitive, social, behavioral, and physical dimensions associated with intensive smartphone use in participants’ accounts.

### Limitations

The findings of this study should be interpreted in light of several limitations. First, participants were recruited from a single university, which limits the transferability of the findings to students from other institutions, regions, age groups, and sociocultural contexts. In addition, the predominance of female participants limited the opportunity to explore possible gender-related differences in smartphone-use experiences. Although variation across departments within the Faculty of Health Sciences was considered during recruitment, participants’ specific departmental affiliations were not systematically recorded; therefore, possible differences in smartphone-use experiences according to academic discipline could not be examined.

Second, the study did not employ a validated measure of problematic smartphone use. The criterion of ≥4 h of daily smartphone use was used only as a purposive sampling criterion to identify individuals with relatively intensive smartphone use and should not be interpreted as a diagnostic or screening threshold for PSU. High usage duration alone does not establish problematic use, and the present study therefore cannot establish participants’ PSU status on the basis of a validated assessment. Similarly, participants’ subjective descriptions of feeling “addicted” or experiencing difficulty controlling their use cannot be interpreted as evidence of smartphone addiction.

Third, social media was the most frequently used smartphone activity for many participants. Consequently, some findings, particularly those concerning algorithms, social feedback, and social comparison, may reflect social-media-specific experiences rather than smartphone use more broadly. Because problematic social media use was not independently assessed, PSU and problematic social media use cannot be empirically disentangled in the present sample. Fourth, the study relied primarily on participants’ self-reported experiences. Information regarding smartphone-use duration, purposes of use, perceived difficulties, and associated emotional and cognitive experiences may therefore not fully correspond to objectively recorded usage behavior. In addition, although the interview guide was pilot tested and open-ended questions and neutral probes were used, the wording and framing of interview questions may still have influenced how participants described or interpreted their smartphone-use experiences.

Finally, the cross-sectional phenomenological design does not permit causal or temporal inferences regarding the relationships among emotional states, temporary relief, social feedback, self-evaluation, routinized use, and other processes identified in participants’ accounts. The proposed process-oriented and potentially cyclical interpretation should therefore be regarded as a hypothesis-generating theoretical interpretation of participants’ lived experiences rather than as an established causal mechanism. Longitudinal and experimental studies are needed to examine whether and how these processes influence one another over time.

### Implications for practice and future research

The findings of the present study have several practical and research implications. First, they suggest that interventions targeting PSU may be insufficient if they focus exclusively on reducing screen time. Because participants described smartphone use as serving psychological functions such as emotion regulation, cognitive escape, and social comparison, intervention programs should also address these underlying psychological processes. Strengthening emotion regulation skills and promoting engagement in meaningful offline activities may represent important strategies for preventing PSU. The findings further indicate that meaningful social relationships, contact with nature, and goal-directed daily activities may naturally reduce smartphone use by providing alternative sources of engagement. Accordingly, psychoeducational programs and preventive interventions implemented in university settings may benefit from encouraging offline social participation and fostering adaptive coping strategies, rather than focusing solely on restricting digital use.

Future research should examine the psychological processes identified in the present study using longitudinal and quantitative research designs to provide a more robust understanding of the mechanisms underlying PSU. In particular, investigating emotion regulation, cognitive avoidance, social comparison, self-regulation, and habit formation within integrated theoretical models may advance the understanding of how these processes jointly contribute to PSU. Furthermore, phenomenological studies involving different age groups and cultural contexts could help clarify how the lived experiences and patterns of smartphone use vary across diverse populations.

## Conclusion

This study provides an in-depth account of how university students experience intensive smartphone use and the aspects of this use that they perceive as difficult to regulate or problematic. Participants’ narratives highlighted interrelated emotional, cognitive, social, and behavioral dimensions, including emotion regulation, cognitive escape, social-media-based feedback and comparison, self-evaluation, and routinized patterns of smartphone use. Considered together, these experiences are compatible with a process-oriented and potentially cyclical interpretation of smartphone use. However, the present phenomenological findings do not establish causal relationships or a temporal sequence among these processes. The proposed cyclical account should therefore be understood as a theoretical interpretation of participants’ lived experiences rather than as an empirically demonstrated mechanism of PSU. Longitudinal and experimental research is needed to determine whether and how these processes interact over time. The findings further suggest that meaningful social relationships, contact with nature, and goal-directed offline activities were experienced by participants as contexts associated with reduced smartphone use. Overall, the study highlights the value of understanding intensive smartphone use in relation to the meanings and functions it holds in individuals’ everyday lives rather than focusing solely on screen time.

## Data Availability

The datasets presented in this article are not readily available because the interview transcripts and micro-journal entries contain potentially sensitive and context-specific information that could compromise participant confidentiality. Public sharing of the raw qualitative data was not covered by the informed consent obtained from participants. Further inquiries regarding the data may be directed to the corresponding author, subject to ethical and confidentiality requirements. Requests to access the datasets should be directed to Tuğba Türkkan, tugba.turkkan@gumushane.edu.tr.
