# Supplementary material for: Exploring the psychological dynamics of intensive smartphone use among university students: a phenomenological study
Source: Front Psychol. 2026 Oct 7;17:1953912. doi: 10.3389/fpsyg.2026.1953912 (PMC13683697; doi:10.3389/fpsyg.2026.1953912)
Supplement: Supplementary file 1 [file Table_1.DOCX]

**Semi-Structured Interview Guide**

**0. Warm-up Question**

**Could you tell me a bit about how you typically use your smartphone during a regular day and what you mostly use it for?**

**Probe:**

- **What role does it play in your daily routine?**

**1. Main Question**

**How would you describe the relationship, if any, between your smartphone use and your mood in everyday life?**

**Probes:**

- **Are there particular situations or contexts in which you notice this relationship more clearly?**
- **Do you notice any differences in these experiences over shorter or longer periods of time?**

**2. Grand Tour Question**

**Could you walk me through a day when you used your smartphone very intensively, describing the day step by step from the time you woke up until you went to sleep?**

**Probes:**

- **How did the day begin?**
- **Were there particular moments when your smartphone use increased or decreased? What was happening at those times?**
- **Were there any moments when your pattern of use noticeably changed?**
- **How were you feeling by the end of the day?**

**3. Main Question**

**Could you similarly describe a day when you used your smartphone considerably less than usual?**

**Probes:**

- **What was different about that day?**
- **What did you notice about your mood?**
- **What was happening in your social or physical environment?**
- **What thoughts, if any, did you have about yourself or your smartphone use?**

**4. Main Question**

**How, if at all, does your smartphone use change during times when you feel stressed or anxious?**

**Probe:**

- **What, if anything, do you look for or experience when you use your smartphone during those moments?**

**5. Main Question**

**When you are away from your smartphone or unable to use it for a period of time, what, if anything, do you notice in terms of your thoughts or feelings?**

**6. Main Question**

**How would you describe your experiences with the content you encounter on social media?**

**Probes:**

- **What, if anything, do you notice about your mood or thoughts about yourself when viewing this content?**
- **Do you notice any differences in the types of content you engage with depending on how you are feeling?**

**7. Main Question**

**Do you ever notice your screen time being higher than usual? If so, what thoughts or feelings, if any, do you have about your smartphone use or yourself during those periods?**

**8. Main Question**

**What does “excessive” smartphone use mean to you? What does “problematic” smartphone use mean to you?**

**Probes:**

- **Do these terms have different meanings for you?**
- **How, if at all, does your own smartphone use relate to these descriptions?**

**9. Main Question**

**Have you ever tried to limit or reduce your smartphone use? If so, could you describe that experience?**

**Probes:**

- **What did you find helpful or unhelpful?**
- **What, if anything, was particularly challenging?**

**10. Closing Question**

**Is there anything else about your smartphone use and daily life that we have not discussed but that you think is important to mention?**
